# Postoperative respiratory state assessment using the Integrated Pulmonary Index (IPI) and resultant nurse interventions in the post-anesthesia care unit: a randomized controlled trial

**DOI:** 10.1007/s10877-020-00564-1

**Published:** 2020-07-29

**Authors:** Suzanne J. L. Broens, Susan A. Prins, Dorinne de Kleer, Marieke Niesters, Albert Dahan, Monique van Velzen

**Affiliations:** 1 Anesthesia and Pain Research Unit, Department of Anesthesiology, Leiden University Medical Center, 2300 RC Leiden, The Netherlands; 2 Department of Anesthesiology, Antoni van Leeuwenhoek Hospital, 1066 CX Amsterdam, The Netherlands; 3 Leiden University Medical Center, Albinusdreef 2, 2333 ZA Leiden, The Netherlands

**Keywords:** Respiratory monitoring, Anesthesia, Opioids, Respiratory depression, Apnea

## Abstract

Although postoperative adverse respiratory events, defined by a decrease in respiratory rate (RR) and/or a drop in oxygen saturation (SpO_2_), occur frequently, many of such events are missed. The purpose of the current study was to assess whether continuous monitoring of the integrated pulmonary index (IPI), a composite index of SpO_2_, RR, end-tidal PCO_2_ and heart rate, alters our ability to identify and prevent adverse respiratory events in postoperative patients. Eighty postoperative patients were subjected to continuous respiratory monitoring during the first postoperative night using RR and pulse oximetry and the IPI monitor. Patients were randomized to receive intervention based on standard care (observational) or based on the IPI monitor (interventional). Nurses were asked to respond to adverse respiratory events with an intervention to improve the patient’s respiratory condition. There was no difference in the number of patients that experienced at least one adverse respiratory event: 21 and 16 in observational and interventional group, respectively (p = 0.218). Compared to the observational group, the use of the IPI monitor led to an increase in the number of interventions performed by nurses to improve the respiratory status of the patient (average 13 versus 39 interventions, p < 0.001). This difference was associated with a significant reduction of the median number of events per patient (2.5 versus 6, p < 0.05) and a shorter median duration of events (62 s versus 75 s, p < 0.001). The use of the IPI monitor in postoperative patients did not result in a reduction of the number of patients experiencing adverse respiratory events, compared to standard clinical care. However, it did lead to an increased number of nurse interventions and a decreased number and duration of respiratory events in patients that experienced postoperative adverse respiratory events.

## Introduction

Several surgery and anesthesia-related factors increase the risk of an adverse respiratory event (ARE) in the perioperative period. Most importantly, AREs are highly associated with the use of opioid therapy for pain, resulting in opioid-induced respiratory depression (OIRD) [1–4]. OIRD is a potentially lethal complication of activation of opioid receptors in brainstem respiratory neuronal network, associated with bradypnea, apnea, hypercapnia and hypoxia. The number of OIRD events in the postoperative period is not known. A recent multicenter observational study showed the occurrence of an OIRD event in 46% of patients in the first 48 h after surgery under general anesthesia [5, 6]. In that study, continuous capnography was used to detect patterns of OIRD. Still, deterioration of the patient from a capnography-related pattern abnormality to a sentinel event requiring an intervention (e.g., administration of opioid antagonist naloxone, reintubation, mechanical ventilation, cardiopulmonary resuscitation, or transfer to the ICU) is much less common and may occur not more than once in every 200–1000 postoperative patients [2, 5, 7]. When respiratory deterioration does occur, however, results can be catastrophic and costs for both the patient and the healthcare system are high [2, 5, 8, 9]. Given the availability of effective interventions, these respiratory catastrophes following routine, elective surgery have been termed ‘never events’ in that they should never be allowed to occur [9].

The challenge is to predict or identify respiratory events and intervene before any further respiratory deterioration. Available scoring systems, such as the STOP-BANG questionnaire, which is based on patient-related risk factors, predict postoperative AREs poorly [10, 11]. Moreover, current standard monitoring practices in postoperative patients do not detect many instances of respiratory compromise [4, 5, 12–14]. Sun et al. showed that prolonged episodes of hypoxemia are common in the first 48 h following non-cardiac surgery and that 90% of these events were missed by routine 4-hourly spot checks in the postoperative wards [15]. Similarly, Lee et al. showed that the time between the discovery of respiratory depression and the last nursing assessment was 2 h in 42% of the cases and a concerning 15 min in 13% of the cases [13].

Given all of the above, continuous respiratory monitoring in patients receiving parenteral opioids in the first 24 postoperative hours has been advocated by multiple stakeholders (including the Anesthesia Patient Safety Foundation, the Joint Commission, the American Society for Pain Management Nursing) [16]. A systematic review of studies evaluating continuous monitoring *via* pulse oximetry or capnography reported improved detection of oxygen desaturation or OIRD-events compared to routine nursing checks [17]. However, impact on clinical outcomes has so far not been demonstrated [12, 17]. Furthermore, continuous monitoring, regardless of which parameter it is based on, has its own limitations, with a potential for false positive alarms disrupting nurse workflow and leading to alarm fatigue [1, 12, 16–18]. Recent developments aim to use multiple parameters to detect AREs. Application of smart algorithms that combine individual physiological variables into one index may increase the ability to detect a true adverse respiratory event while avoiding false alarms and limiting alarm fatigue [1]. An example of such a multiparameter index is the Integrated Pulmonary Index or IPI™, which integrates oxygen saturation (SpO_2_), respiratory rate (RR), end-tidal PCO_2_ (P_ET_CO_2_) and heart rate (HR) into a single integer value of 1–10 that represents adequacy of respiratory condition of the patient using a fuzzy logic inference mathematical model [18, 19]. So far, the IPI has been validated with retrospective data obtained in a variety of clinical settings, but it has not been studied prospectively as a monitor of postoperative AREs [19].

In this randomized controlled trial, the use of the IPI was compared to standard continuous respiratory monitoring in postoperative patients. Our aim was to determine whether the IPI enables early detection of postoperative respiratory events and alters clinical interventions.

## Materials and methods

Initially we performed an observational trial to evaluate the clinical utility of the IPI algorithm in postoperative patients and to determine the incidence of AREs. The results of this study are published elsewhere [20]. The data generated by this study were used to design and power the current study.

### Ethics and patients

The protocol was approved by the Investigational Review Board (IRB) (Commissie Medische Ethiek, Leiden University Medical Center, the Netherlands) in August 2015. All study procedures were performed in compliance with the 2013 version of the Declaration of Helsinki and Good Clinical Practice guidelines. The study was registered at the trial register of the Dutch Cochrane Center under identifier 5231. Patients were recruited between November 2017 and January 2019. Subjects were enrolled for the study and they gave verbal and written informed consent prior to study procedures.

### Patients

 Patients were adult (at least 18 years old), American Society of Anesthesiologists (ASA) class 1–3, scheduled for elective surgery under general anesthesia, expected to receive opioids for treatment of postoperative pain, and requiring an overnight post-anesthesia care unit (PACU) stay following surgery. Exclusion criteria included use of epidural anesthesia, nerve blocks, surgery that would hamper the postoperative application of the IPI sensors, emergency surgery or the inability to give informed consent.

### Study design

The study had a two-arm, parallel, randomized controlled design. Patients were randomized on the day of surgery to an observational arm or an interventional arm using a computer-generated randomization list. Neither patient nor the anesthetic team responsible for clinical care during surgery were informed of the allocation. Due to the nature of the study, patients and PACU nurses were not blinded to the study allocation once data collection commenced. Outcome assessors were blinded to allocation.

#### Clinical care in both treatment groups

Anesthesia technique (total intravenous anesthesia or volatile anesthesia, opioid use, use of neuromuscular reversal agents) was left to the discretion of the attending anesthesiologist. Once surgery had ended and the patient was extubated and transported to the PACU, the patient was connected to standard monitoring equipment (3-lead ECG, non-invasive blood pressure monitoring using an arm cuff, pulse oximetry *via* a finger probe). Additionally, the standalone Capnostream 20p monitor (Medtronic, Fridley, Minnesota) was connected to the patient. This monitor collects SpO_2_ and HR measurements *via* pulse oximetry using a finger probe. Additionally, it monitors P_ET_CO_2_ and RR *via* a nasal cannula that allows oral and nasal sampling of inspired and exhaled air as well as the delivery of supplemental oxygen with flow rates up to 5 L/min (FilterLine®, Medtronic). All patients were admitted to the PACU until 8AM the following morning, when monitoring by Capnostream was discontinued. Any complication or the need for a prolonged PACU stay was noted in the patient electronic health record. Nurses were asked to note instances in which the Capnostream monitor (finger probe or nasal cannula) was disconnected as may have occurred during meals or patient care.

#### Clinical care in the observational arm

Patients randomized to the observational arm of the study were attached to the Capnostream monitor, but the monitor screen was shielded and Capnostream alarms were silenced. Nurses were instructed to treat their patients according to standard clinical care using standard monitors and clinical experience. The PACU nurses were requested to note every respiratory event such as apnea, hypoxia, respiratory depression or obstructed breathing. For every respiratory event, they were also asked to note the associated intervention to improve respiratory condition such as verbal or tactile patient stimulation, chin lift, administration of supplemental oxygen *via* the nasal cannula, escalation to involve the attending PACU physician, naloxone administration, or reintubation. The decision to intervene and manner of intervention was based on local protocol, which relies on monitoring of SpO_2_, respiratory rate and sedation level.

#### Clinical care in the interventional arm

Patients randomized to the interventional arm of the study were attached to the Capnostream monitor with the screen visible to the nursing staff. The screen displays the capnography trace (including actual P_ET_CO_2_ values), HR, RR, SpO_2_ and IPI value. Prior to the start of the study, nurses were trained to use the Capnostream monitor and interpret the IPI values. A prestudy run in 29 patients was performed (results not included) to finetune the set-up and feasibility of the protocol. Based on the prestudy run, the alarm threshold was set to an IPI value of 1 prior to start of the study.

Since it is mandatory in our hospital to collect vital signs in the patient’s electronic health record, the patient was also attached to standard clinical monitors. However, nurses were requested to guide their assessment of the patient’s respiratory condition based on the IPI value. In case of a clinically relevant discrepancy between the monitors, nurses were asked to evaluate the patient and intervene according to their experience and report the discrepancy to the investigators. In case of an alarm at an IPI value of 1, the nurses were instructed to approach the patient, assess the patient’s condition (apnea, respiratory depression, hypoxia, but also whether the low IPI event could be considered an artefact) and intervene as required (see above). The occurrence of a low IPI event was noted as well as the assessment of the patient’s condition.

### Data collection

Data were collected from the Capnostream monitor, the electronic health record database (Healthcare Information X-change (HiX), Chipsoft, the Netherlands) and the case record forms (CRF) containing the notes regarding respiratory events, and interventions. The Capnostream monitor stored data at 0.5 Hz intervals; the HiX database provided information regarding patient history and characteristics, and drugs administered during surgery and during PACU stay. Major complications were recorded in the electronic health record and the CRF, adverse events were collected in the CRF.

#### Primary and secondary study endpoints

The primary study endpoints were the number of low IPI events and the number and nature of the nurse responses to low IPI values. Secondary endpoints were the duration of IPI events and the main causes of IPI events, as determined by one or more of the 4 individual variables used in the calculation of the IPI value.

#### Data selection

We manually checked the data for the presence of artefacts at the end of the study. Low IPI events were considered true and clinically relevant adverse respiratory events if (1) the nurse had not noted the event as an artefact or sensor mispositioning and (2) the recording of vital signs from the electronical medical database corroborated the Capnostream data and (3) in case of hypoxia or obstructed breathing (as noted by the nurses), the event was followed by a sympathetic response such as tachycardia. Once a low IPI event was confirmed to be a true ARE, the nature of the event was examined. An event was considered to be associated with hypoxia when the SpO2 was ≤ 90%; an event was considered a respiratory depression event when: (1) RR < 6 breaths/min or (2) at least 1 episode of apnea (RR = 0 breath/min for at least 15 s) and (1) end-tidal PCO_2_ > 60 mmHg) or (2) end-tidal PCO_2_ < 15 mmHg.

### Statistical analysis

We assumed that an intervention would be required in all subjects of the interventional study, and assuming an ‘intervention:low IPI event’ ratio of 99.9% in the interventional arm and 70% in the observational arm, 35 patients were required per group to assess if IPI based intervention differs from local protocol (alpha = 0.05, 1-beta = 0.9). Because of anticipated drop-outs, we aimed to include 80 patients in the study (40 per group).

The normal distribution of numerical data was visually assessed and groups were subsequently compared using either independent samples t-tests or Mann-Whitney U tests. Categorical data were compared using Pearson’s chi-squared test. Results were considered significant with a p-value of < 0.05. Statistical analyses were performed using IBM SPSS statistics for Windows v25.0 (IBM Corp., Armonk, NY).

## Results

**Fig. 1:**
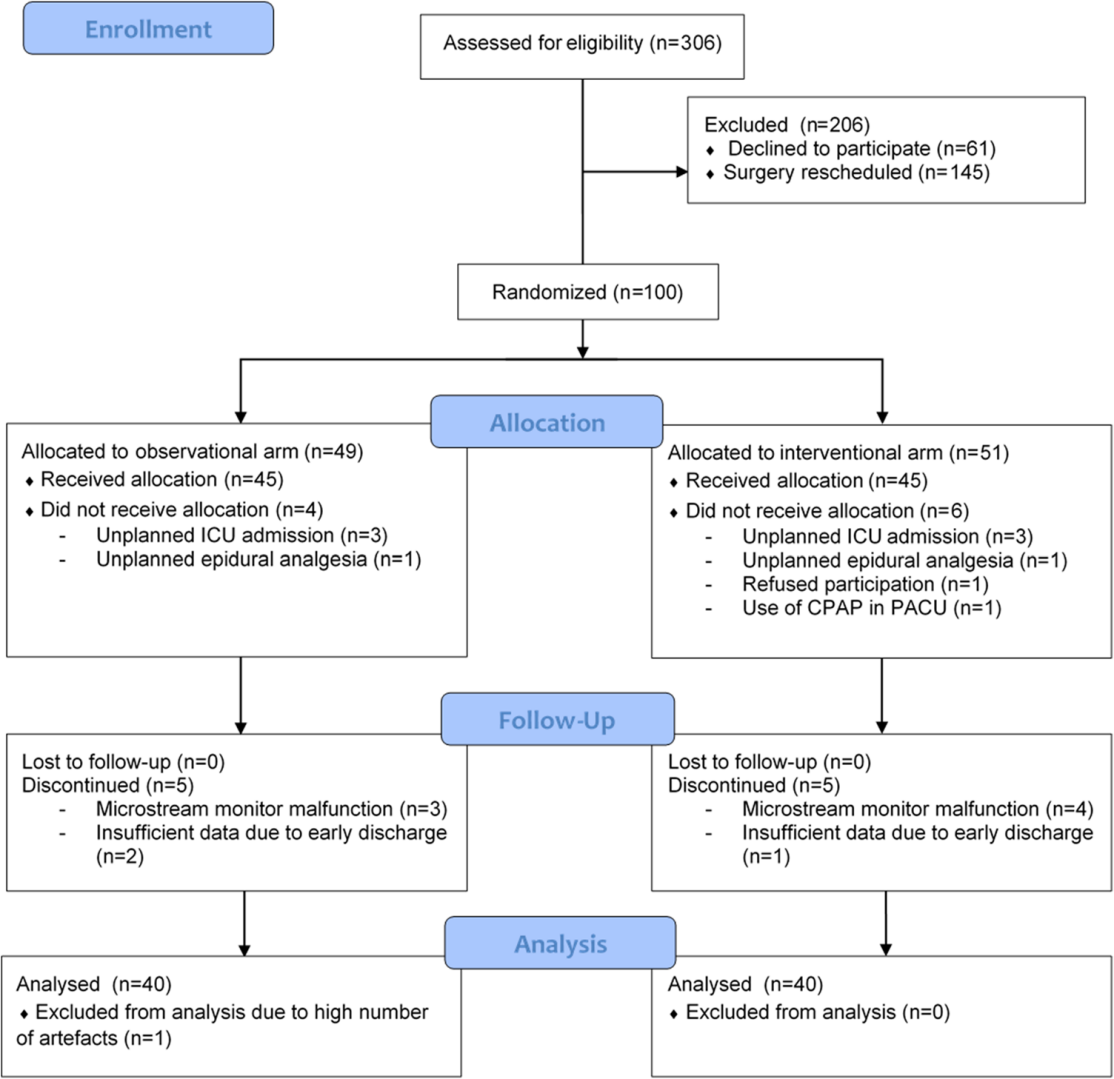
Study flow chart. *CPAP* continuous positive airway pressure, *ICU* intensive care unit, *PACU* post-anesthesia care unit

A total of 306 patients were approached for participation. 206 patients either refused participation (n = 61) or were not randomized because their surgery was rescheduled (n = 145). During the inclusion period, logistic issues (mostly shortage of PACU bed availability) caused many surgeries to be rescheduled. One hundred patients were randomized, of which 20 did not complete the study. The reasons for dropout or exclusion are given in the Fig. 1. Eighty patients completed the study, 40 in the observational arm and 40 in the interventional arm. In the observational arm, one patient was excluded from analysis due to an exceptionally high number of artefacts in the Capnostream monitor data. The data of 79 patients were included in the analysis.

### Patient characteristics

Patient and peri-operative characteristics are given in Table 1. Both groups were similar in terms of age, ASA classification and risk factors for postoperative respiratory events. A total of 28 patients (35%) had a STOP-BANG of > 3 and were considered at high risk for the presence of obstructive sleep apnea (OSA). Nine other patients had been diagnosed with OSA, of which one used a continuous positive airway pressure device at home. In 38% of patients, there was no pre-operative risk assessment for the presence of OSA. Seventy-six patients (95%) received either morphine or methadone 45 to 60 min before the end of surgery to cover the initial part of postoperative analgesia. During their PACU admission, fifty-two (66%) patients required additional intravenous opioids (morphine, methadone or fentanyl); 19 (24%) patients received either morphine or fentanyl by a patient-controlled analgesia (PCA) device. Twenty-seven (34%) patients did not require additional opioids postoperatively. Five of these patients had received an intraoperative continuous sufentanil infusion for more than 6 h, the remainder had been given either morphine or methadone during surgery.

**Table 1:** Patient and peri-operative characteristics

	Observational arm	Interventional arm	Standardized mean difference
Number of patients	39	40	
Sex (male/female)	24/15	22/18	
Age (years), average (SD)	54.3(14.6)	59.2(11.8)	0.37
BMI (kg/m^2^), average (SD)	26.7(4.7)	26.2(3.7)	0.13
BMI ≥ 30, n (%)	9(23)	6(15)	
ASA Class, n (%)			
I	3(7.7)	3(7.5)	
II	17(43.6)	13(32.5)	
III	19(48.7)	24(60.0)	
Comorbidities, n (%)			
Cardiac	10(26)	11(28)	
Pulmonary	5(13)	4(10)	
Diagnosed with OSA, n (%)	4 (10)	5 (13)	
Requiring home CPAP, n	0	1	
STOP-BANG scores, n (%)	11(28)		
Above 3	16(41)	17(43)	
Below or equal to 3	16(41)	14(35)	
Unknown	12(31)	9(23)	
Type of surgery, n (%)			
General abdominal	23(59)	26(65)	
Vascular	4(10)	6(15)	
Reconstructive	5(13)	5(13)	
Orthopedic	3(8)	0(0)	
Neurosurgical	2(5)	1(3)	
Head and neck	2(5)	2(5)	
Duration of surgery (hours), average (SD)	3.8(2.1)	3.2 (2.1)	0.29
Intra-operative opioid loading dose (mg), average (SD)	11.3(3.7)	11.0 (3.1)	0.30
No intra-operative opioid loading dose, n	1	3	
Postoperative opioid analgesia, n (%)			
None	10(26)	17(43)	
Patient-controlled analgesia	12(31)	7(18)	
Capnostream IPI collection time (hours), average (SD)	15.8 (3.3)	16.2(3.9)	0.11

For continuous variables, the (absolute) standardized mean difference is listed in the right column

*ASA* American Society of Anesthesiology, *BMI* body mass index, *CPAP* continuous positive airway pressure, *IPI* integrated pulmonary index, *n* number, *OSA* obstructive sleep apnea, *SD* standard deviation, *STOP-BANG* snoring-tired-observed-pressure BMI-age-neck-gender, questionnaire covering several risk factors related to obstructive sleep apnea

### Adverse respiratory events and artefacts

#### ***Incidence***

A total of 860 low IPI events were recorded, of which 523 (61%) were considered artefacts and 337 were considered true adverse respiratory events. The proportion of artefacts was similar in both groups (60% in the observational group *versus* 64% in the interventional group, p = 0.292). In 37 (47%) patients, one or more true respiratory events were recorded, 21 in the observational group and 16 in the interventional group (p = 0.218). The number of respiratory events was almost four times higher in the observational group than in the interventional group (265 *versus* 72 respectively), accounting for 79% of all events.

In patients experiencing at least one ARE, those in the observational group experienced a median of 6 events compared to 2.5 events for those in the interventional group (p < 0.05). When corrected for length of PACU stay, patients in the observational group experienced 0.4 events/h compared to 0.1 events/h in the interventional group (p < 0.05). Moreover, the median duration of events was significantly longer in the observational arm than in the interventional arm (75 s *versus* 62 s, respectively, p < 0.001). The increased incidence and duration of true respiratory events in the observational group is reflected in the total time spent in a state of potential respiratory compromise: 57 s/h of PACU admission time in the observational group compared to 9 s/h of PACU admission time in the interventional group (p < 0.05). These findings are summarized in Table 2.

**Table 2:** Characteristics of adverse respiratory events

	Observational arm	Interventional arm	
Adverse respiratory events, n	660	200	
True event / artefact	265 / 395	72 / 128	p = 0.292*
Patients with at least one event, n (%)	21(54)	16(40)	p = 0.218*
*In patients with ≥ 1 event*			
Events per patient (n), median (range)	6(1–37)	2.5(1–17)	p < 0.05#
Events per hour (n), median (range)	0.4(0.06–2.53)	0.1(0.05–1.44)	p < 0.05#
Duration of event (sec), median (range)	75(29–848)	62(28–424)	p < 0.001#
Time spent in true ARE (sec/h), median (range)	57(2-381)	9(2–88)	p < 0.05#
Characteristics of ARE, n (%)			p < 0.001*
Respiratory depression without hypoxia	231(87)	47 (65)	
Respiratory depression with hypoxia	34(13)	21(29)	
Hypoxia without respiratory depression	0(0)	4(6)	

An adverse respiratory event (ARE) is defined as an Integrated Pulmonary Index (IPI) of 1 for at least 30 s. Events were considered true if the nurse did not annotated the event as an artefact/sensor mispositioning, if the vital signs recording of the electronic medical record corroborated with the findings, and if the event was followed by a sympathetic response. Respiratory depression was defined as respiratory rate < 6 breaths per minute or at least 1 episode of apnea and end-tidal PCO_2_ > 60 mmHg, or end-tidal PCO_2_ < 15 mmHg. Hypoxia was defined as SpO_2_ ≤ 90%

*ARE* adverse respiratory event, *h* hour, *IPI* integrated pulmonary index, *n* number, *sec* seconds

*Pearson’s Chi-square or Fisher’s Exact test

#Mann-Whitney U-test or independent t-test, depending on data distribution

#### Respiratory depression and hypoxia

Only 18% (59/337) of all respiratory events were associated with hypoxia. Most events were episodes of respiratory depression with (n = 55 total, 16%) or without (n = 278, 82%) hypoxia. All alarms associated with respiratory depression were caused by low RR with low P_ET_CO_2_. Hypercapnia did not trigger any alarms, as the highest P_ET_CO_2_ in our population was 56 mmHg. The proportion of respiratory events associated with hypoxia was significantly higher in the interventional group than in the observational group. These findings are summarized in Table 2.

### Interventions

A total of fifty-two interventions were reported by the nurses, of which 39 occurred in the interventional arm and 13 in the observational arm of the study. Interventions were mostly limited to verbal or tactile stimulation of the patient and/or initiating or increasing supplemental oxygen administration. A list of all interventions is found in Table 3.

**Table 3:** Characteristics of respiratory interventions by PACU nurses

	Observational arm	Interventional arm	Total
Interventions, n	39	13	52
Interventions in the absence of an ARE, n	8	15	23
*Intervention*, n			
Verbal or tactile stimulation	5	21	26
Increase supplemental O_2_	8	8	16
Decrease supplemental O_2_	0	2	2
Patient repositioning	0	1	1
Combination of the above	0	2	2
No intervention required*	0	5	5

Respiratory interventions as reported by the PACU nurses

*ARE* adverse respiratory event, *n* number

*In 5 respiratory events, no intervention turned out to be required as the patient recovered spontaneously or the Capnostream ‘low IPI’ alarm sound aroused the patient resulting in improvement of respiration

Of the 52 interventions, 23 were initiated by the nurses despite the absence of a respiratory event as defined by an IPI of 1 for more than 30 s (8 in the observational arm and 15 in the interventional arm). Interventions by nurses for these non-low IPI events were initiated either by hypoxia that was not low enough to trigger an IPI alarm (e.g. increasing oxygen administration when SpO2 was < 94% but > 91%) or by episodes of apnea < 30 s. The ratio of respiratory interventions to respiratory events was significantly lower in the observational group (13/265) than the interventional group (39/72) (p < 0.00001).

#### Serious adverse respiratory events

Despite the occurrence of respiratory events, there was no need for naloxone administration, airway maneuvers or assisted ventilation in either group. However, two patients had an abnormal postoperative course related to respiratory complications. In one case a pulmonologist was consulted on the day after surgery for persisting hypoxia and high oxygen requirement. This patient had been randomized to the interventional arm of our study, had triggered 17 IPI alarms and had received 9 interventions (tactile stimulation and supplemental oxygen administration). The consultation did not delay discharge from the PACU. The patient was subsequently diagnosed with emphysema and followed-up in an outpatient setting. In a second patient that had been randomized to the observational arm of the study, a routine morning arterial blood gas revealed an acute respiratory acidosis, which was considered to be opioid-induced and for which PACU discharge was delayed by 24 h. In this patient, the IPI monitor had recorded 29 events of respiratory depression with signs of obstructed breathing. However, the nurses had only noted one respiratory event and intervened by increasing the supplemental oxygen administration.

### Risk factors for respiratory events

When comparing characteristics for the patients that experienced AREs as opposed to none, only sex was significantly different, with male patients being more likely to experience a respiratory event. Other known risk factors were not significantly different between groups (see Table 4). The average cumulative postoperative opioid dose did not differ significantly between patients with or without adverse respiratory events (6.6 mg vs. 5.1 mg, respectively; p = 0.31).

**Table 4:** Risk factors for adverse respiratory events

	Without adverse respiratory events	With adverse respiratory events	
Number of patients, n (%)	42(53)	37(47)	
Sex (male/female)	18/24	28/9	p < 0.01*
Age (years), average (SD)	56.4(13.1)	57.1(13.9)	p = 0.8#
BMI (kg/m^2^), average (SD)	26.5(4.5)	26.4(3.9)	p = 0.94#
BMI ≥ 30, n (%)	9(21)	6(16)	p = 0.58*
ASA Class, n (%)			
I	5(12)	1(3)	p = 0.13*
II	18(43)	12(32)	
III	19(45)	24(65)	
Comorbidities, n (%)			
Cardiac	10(24)	11(30)	p = 0.61*
Pulmonary	3(7)	6(16)	p = 0.29*
Diagnosed with OSA, n (%)			
Requiring home CPAP, n	4(10)	5(14)	p = 0.72*
STOP-BANG scores, n (%)	0	1	p = 0.87*
Above 3	14(33)	14(38)	
Below or equal to 3	17(40)	13(35)	
Unknown	11(26)	10(27)	
Duration of surgery (hours), average (SD)	3.7(2.4)	3.3(1.6)	p = 0.35#
Intra-operative opioid loading dose (mg), average (SD)	10.2(4.1)	11.1(4.2)	
No intra-operative opioid loading dose, n	3	1	p = 0.33#
Postoperative opioid analgesia, n (%)			
None	16(38)	11(30)	p = 0.48*
Patient-controlled analgesia	11(26)	8(22)	p = 0.79*
Postoperative cumulative opioid dose (mg), average (SD)	5.1(6.6)	6.6(6.1)	p = 0.31#

*ASA* American Society of Anesthesiology, *BMI* body mass index, *CPAP* continuous positive airway pressure, *n* number, *OSA* obstructive sleep apnea, *SD* standard deviation, *STOP-BANG* snoring-tired-observed-pressure BMI-age-neck-gender, questionnaire covering several risk factors related to obstructive sleep apnea

*Pearson’s Chi-square or Fisher’s Exact test

#Mann-Whitney U-test or independent t-test, depending on data distribution

## Discussion

To our knowledge, this is the first randomized controlled trial that studied the use of a multiparameter monitoring system to track the respiratory status of postoperative patients. We found that compared to continuous monitoring using respiratory rate and pulse oximetry alone, the use of the IPI monitor led to an increase in the number of interventions performed by nurses to improve the respiratory condition of the patient. This did not lead to a reduction in the number of patients that experienced an ARE, but did cause a significant reduction of the number of events per patient combined with a shorter duration of respiratory events.

Our study shows that 47% of patients experienced one or more adverse respiratory events in the first 24 h following surgery, with an equal distribution among randomization arms. This is consistent with the findings in the PRODIGY trial [5]. In that prospective, observational, multicenter trial, patients receiving opioids for postoperative pain relief were monitored with the Capnostream monitor and separate signals (end-tidal CO_2_, RR, SpO_2_ and HR) were collected and analyzed. Respiratory depression was defined by an end-tidal PCO_2_ ≤ 15 or ≥ 60 mmHg for at least 3 min, RR < 6 breaths/min for at least 3 min, apnea lasting more than 30 s, or any opioid-related adverse respiratory event. A respiratory depression event occurred in 46% of 1,335 patients.

The clinical relevance of many of these events remains unknown, as deterioration towards a serious adverse event requiring major interventions was fortunately infrequent in our study. However, both studies suggest that nearly half of our patients spend some of the early postoperative period in an state of potential respiratory compromise.

Aside from male sex, we did not detect any risk factors for the occurrence of a postoperative AREs. Other studies, including the aforementioned PRODIGY trial, detected several predictors of a respiratory event, including age, sleep disorders, opioid naivety or high blood pressure [5, 6, 14]. Our relatively small sample size precluded detection of additional risk factors.

Although monitoring of the IPI did not lead to a reduction in the number of patients experiencing a respiratory event, the number and duration of events per patient was decreased. This is not surprising. Respiratory monitoring *per se* does not affect the propensity of a patient to experience adverse respiratory events, however, when a respiratory alarm leads to a nurse intervention, a cascade of events is interrupted that might otherwise have led to more frequent or prolonged events. Since we allowed use of supplemental oxygen, low SpO_2_ values contributed to the IPI alarm in only 17% of events. In all other cases the events were triggered by low respiratory rate (or apnea). It is important to realize that supplemental oxygen may mask or may even exacerbate opioid induced respiratory depression when only SpO_2_ is monitored [4, 18, 21]. Patients on oxygen will have some oxygen reserve causing a delay in the detection of an obstructive or central apneic event. Additionally, the peripheral respiratory drive is blunted by supplemental oxygen which will cause a further depression of ventilation [21]. Our study indicates that it is a challenge for nurses to identify patients experiencing opioid induced respiratory depression in the absence of hypoxia, even with continuous monitoring of respiratory rate in a high care setting with experienced nurses and a low (1:2) nurse to patient ratio. This suggests that continuous monitoring of just SpO_2_ is not effective for the identification of opioid-induced respiratory depression.

After a prestudy run we decided to put the IPI alarm threshold at 1, because many of the alarms caused by an IPI of 1–4 represented subtle ventilatory disturbances that were clinically irrelevant. As a consequence, the majority of the alarms in our study were caused by low RR or apnea. It is therefore unclear whether IPI-based monitoring confers a benefit over monitoring of respiratory rate alone. The difference lies in the fact that with simple continuous RR monitoring, the alarm threshold is fixed (e.g. ≤5 breaths/min) whereas with a fuzzy logic algorithm, a RR of 5 breaths/min can trigger an alarm when SpO2 is 92% but not when SpO2 is 100%. It would be interesting to compare IPI to continuous respiratory rate monitoring alone in a future study.

We observed a large number of IPI alarm artefacts. These artefacts were related to the mispositioning of the nasal sensor causing sensing artefacts or the result of minor clinical events. One such frequent alarm that we considered an artefact was related to mildly obstructed breathing during sleep which caused low end-tidal PCO_2_ values with low but otherwise normal breathing. Still, while our study suggests that lowering the triggering alarm to an IPI value of 1 can be done safely (no serious respiratory events were missed by the monitor), more than half of the alarms did not represent actual respiratory compromise. For successful implementation of future monitoring systems without disrupting nurse workflow, the issue of false alarms and alarm fatigue needs to be addressed. Interestingly, in case of a true alarm, some of our patients did not require actual interventions because their bedside monitor alarm had aroused them before the nurse had intervened. It is imaginable that future monitoring systems could trigger bedside alarms first and only be transmitted to a nursing pager when the respiratory compromise persists or worsens.

This study has some methodological issues that warrant comment. First, we tested the IPI in the PACU setting. Consequently, extrapolation of our results to the general surgical ward should be done with caution. We chose the current approach in order to have two comparable arms, one arm with intervention based on the IPI monitor, and one arm with intervention based on standard PACU monitors (RR and SpO_2_) allowing reliable comparison of event occurrences and interventions. We observed that patients receiving respiratory intervention based on the IPI monitor had less and shorter adverse respiratory events, compared to control patients. We expect the benefit of the IPI monitor to be higher on the general surgical ward, where opioids are given, respiratory events can occur but clinical monitoring is infrequent[22].

Second, we studied the IPI monitor in a population of patients expected to require opioids in the first 24 h after surgery. Several studies have shown that events are most likely to occur within this timeframe and that the use of opioids is a major risk factor for adverse respiratory events [1–3, 13]. However, half of our patients did not experience any respiratory event and not all patients received an opioid postoperatively. It is likely that more benefit and less harm (e.g., disruptions because of alarm artefacts) could be achieved if the monitor was tested in a population at intrinsically higher risk for postoperative respiratory events (e.g. patients receiving parenteral opioids).

Given all of the above, we suggest that future studies of continuous respiratory monitoring focus on devices using algorithms that rely on multiple parameters and that most benefit is to be expected in general surgical wards in patients that are expected to be at a high risk of AREs.

In conclusion, the use of the IPI monitor in postoperative patients did not result in a reduction of the number of patients experiencing adverse respiratory events, compared to standard clinical care. However, use of the IPI monitor did lead to an increase in the number of nurse interventions and a decrease in the number and duration of respiratory events in patients that experienced postoperative AREs.
